# Functional connectivity in *in vitro* neuronal assemblies

**DOI:** 10.3389/fncir.2015.00057

**Published:** 2015-10-07

**Authors:** Daniele Poli, Vito P. Pastore, Paolo Massobrio

**Affiliations:** Department of Informatics, Bioengineering, Robotics and System Engineering, University of GenovaGenova, Italy

**Keywords:** functional connectivity, correlation, neuronal network dynamics, *in vitro*, graph theory, micro-electrode arrays

## Abstract

Complex network topologies represent the necessary substrate to support complex brain functions. In this work, we reviewed *in vitro* neuronal networks coupled to Micro-Electrode Arrays (MEAs) as biological substrate. Networks of dissociated neurons developing *in vitro* and coupled to MEAs, represent a valid experimental model for studying the mechanisms governing the formation, organization and conservation of neuronal cell assemblies. In this review, we present some examples of the use of statistical Cluster Coefficients and Small World indices to infer topological rules underlying the dynamics exhibited by homogeneous and engineered neuronal networks.

## Introduction

One of the most fundamental features of a neural circuit is its connectivity since the single neuron activity is not due only to its intrinsic properties but especially to the direct or indirect influence of other neurons (Makarov et al., 2005). As recently reviewed by Yuste (2015), the *era* of the “neuron doctrine” has faded: thanks to the technological improvements of the multi-unit recordings, neuronal assemblies can be considered the physiological units of the brain which generate and sustain the functional properties as well as the dynamical states of the entire system. Nervous systems are complex networks *par excellence*, capable of generating and integrating information from multiple external and internal sources in real time. Neural networks in the brain should comply with two competing demands, which might also be considered as fundamental organizational principles: *functional segregation* and *functional integration*, enabling both the rapid extraction of information and the generation of coherent brain states (Sporns et al., 2000). As confirmed by recent studies reporting structural analyses of brain networks carried out on datasets describing the cerebral cortex of mammalian animal models (e.g., rat, cat, monkey), cortical areas were found to be neither completely connected with each other nor randomly linked; instead, their interconnections show a specific and intricate organization (Sporns, 2011). These dynamic interactions were extensively studied by Friston and colleagues in 1994 who emphasized the need to distinguish between *functional* and *effective* connectivity (Friston, 1994). Functional connectivity refers to the correlation between time series from different neurons without any underlying causal model; by contrast, the effective connectivity refers to the direct influences that one neuronal system exerts on another, relying on a network model in which different populations appear structurally connected. During the last years, graph theory and statistical physics provided a valuable contribution to map the functional links extracted directly from multiple brain areas by means of their electrophysiological recordings analysis (Sporns, 2002).

Indeed, the possibility to use a valuable but at the same time reduced and simplified experimental model to understand the functional properties of neuronal networks has been a great breakthrough. Nowadays, dissociated neuronal cultures coupled to Micro-Electrode Arrays (MEAs) are widely used to better understand the complexity of brain networks. In addition, the use of dissociated neuronal assemblies makes possible to manipulate and control their connectivity: in other words, it is feasible to drive the connectivity of a network and to study how such a topological configuration can shape the emergent dynamics. Examples of engineered networks started in 1975 with the pioneering work of Letourneau (1975) who investigated the role of different adhesion substrates for promoting the initiation, elongation and branching of the axons. A great advancement toward the possibility to design *ad hoc* neuronal circuits occurred after the work of Kleinfeld and coworkers who used photoresist technology to pattern hydrophobic and hydrophilic materials for controlling neuronal cell attachment (Kleinfield et al., 1988). More recently, by exploiting the advances in the technology, it has been possible to design and build engineered networks: in 2007, Macis et al. (2007) realized a micro-drop deposition system which guaranteed the controlled deposition of micro-islands of neurons in correspondence of the microelectrodes. Following a similar approach in 2012, Marconi et al. (2012) coupled a few neurons to one microelectrode of a MEA, by designing a sort of regular lattice. More recently, following the idea that the brain has a modular structure, several attempts have been done to recreate *in vitro* interconnected neuronal assemblies (Kanagasabapathi et al., 2011; Levy et al., 2012; Bonifazi et al., 2013; Pan et al., 2015).

In this work, we will review and present to a broad readership, the commonly used approaches to estimate functional connectivity in dissociated networks, and which kind of network topologies modulate the dynamics of dissociated neuronal ensembles coupled to MEAs. After a brief description of the commonly used algorithms and of the metrics used to characterize the connectivity maps, we will describe some significant results considering both the spontaneous and stimulus-evoked activity of homogeneous as well as engineered neuronal networks.

## The use of micro-electrode arrays for inferring functional connectivity

**Figure 1:**
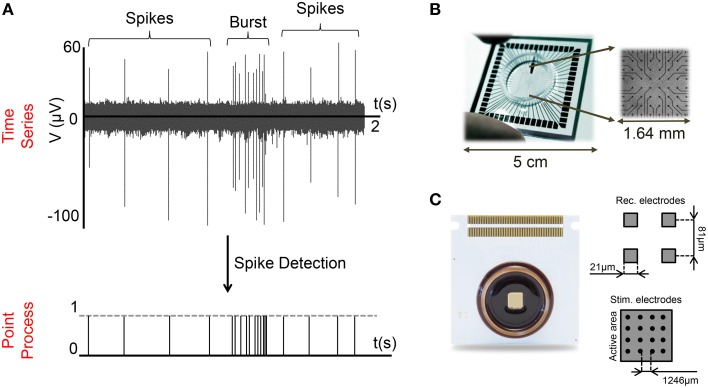
**MEA and extracellular signals. (A)** The activity of a cortical neural network (28 DIVs) presents a mix of bursting and spiking activity (top). Applying a spike detection algorithm, time series are converted into a serial point process (bottom). **(B,C)** Examples of Micro-Electrode Arrays (MEAs) made up of **(B)** 60, **(C)** 4096 electrodes.

MEAs are a powerful tool for simultaneously monitoring and acquiring the electrophysiological activity of neural preparations at many sites (Figures 1A,B). The electrodes embedded in such devices can record electrophysiological activity in a non-invasive way (i.e., extracellularly) and therefore, under proper maintenance conditions, can allow long-term recordings (i.e., from hours up to months) (Potter and DeMarse, 2001). Currently, commercial available MEAs usually provide 60–120 electrodes with 100–500 μm inter-electrode spacing (Figure 1B), or high-density configurations with thousands of microelectrodes (4000–10,000) with a spatial resolution of some tens of micrometers (Figure 1C; Berdondini et al., 2009; Frey et al., 2009).

The characteristics of these devices allow different studies on neuronal networks like electrical (Wagenaar et al., 2005) and chemical manipulation (Pancrazio et al., 2003), and/or physical segregation in sub-populations (e.g., Levy et al., 2012).

More recently the scientific community is beginning to use MEAs for characterizing the underlying functional connectivity, and its interplay with the expressed dynamics (Massobrio et al., 2015b), especially by exploiting the high-density systems which allow a more accurate reconstruction of the network topology (Maccione et al., 2012). The inferred functional networks are “translated” into simple graphs in which the nodes are the neurons, and the links are the connections among the cells. The following methodological sections will briefly present some of these basic measures and will define some strategies aimed at identifying functional connectivity in neuronal assemblies.

## Graph theory

Graphs are made up of nodes which represent the neurons and edges which model the connections (morphological or functional) among the neurons. If we consider the directionality of the connection (i.e., from a pre- to a post-synaptic neuron), the graph is named *directed*, otherwise it is called *undirected*. The structure of the graph is described by the *adjacency matrix* [often named connectivity matrix (CM)], a square symmetric matrix of size equal to the number of nodes *N* with binary entries. If the element *a*_*ij*_ = 1, a connection between the node *j* to *i* is present, otherwise *a*_*ij*_ = 0 means the absence of connections.

To allow a mathematical analysis, the graph, and consequently the network topology, can be characterized by a large variety of parameters (Rubinov and Sporns, 2010). In the field of neuronal networks, the simplest metrics which allow to have a simple but clear indication of the kind of underling connectivity are the Node Degree, the Cluster Coefficient and the Average Path Length (Sporns et al., 2000) which will be briefly described below.

**Figure 2:**
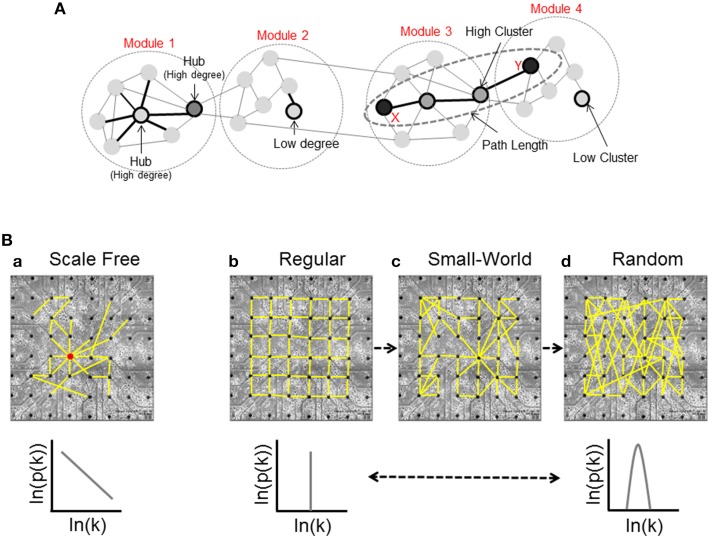
**Basic graph measures and network structures. (A)** Node degree is the number of connections of a given node; this panel shows a simple network divided in four different modules: Module 1, in which we can see a high-connected unit called *hub*, and Module 2, that presents a low connectivity case. Modules 3 and 4 show two units with high and low values of Cluster Coefficient respectively, and an example of shortest path length; the nodes X and Y are connected by the shortest possible path (three links), and two different units that we call *intermediaries*. **(B)** Classification of the network structure [*scale-free* **(a)**, *regular* **(b)***, small-world* **(c)**, and *random* **(d)**] and corresponding degree distributions.

**Node Degree**: the in-degree (*id*) and the out-degree (*od*) of a single node are defined as the number of incoming (afferent) and outcoming (efferent) edges respectively, and the total degree (*td*) is their sum (Figure 2A, Modules 1 and 2).

(1) $$\begin{array}{l} td=id+od \end{array}$$

High in-degree values indicate neural units influenced by a larger number of nodes, while high out-degree values show a large number of dynamic sources. Depending on the node degree distribution, we can identify three stereotyped graphs: scale-free, regular, and random (Figure 2B).

*Scale-free networks* (Figure 2Ba) (Barabasi and Bonabeau, 2003) are characterized by high-connected units called hubs. Hubs are nodes with a degree at least one standard deviation above the network mean. Thanks to this peculiarity, hubs play a significant role on the neural dynamics (Sporns et al., 2007). In the scale-free network, the probability that a generic node *i* has *k* connections is given by a power law relationship:

(2) $$\begin{array}{l} p\left(k\right)\propto k^{-\gamma } \end{array}$$

where γ is the characteristic exponent which ranges experimentally from 1.3 (slice recordings, Bonifazi et al., 2009) to 2 (fMRI recordings, Eguíluz et al., 2005).

*Regular networks* (Figure 2Bb) are ordered and characterized by high segregation values. The integration level of the network grows by increasing the number of graph units. In this case, the probability that *i* has *k* connections is given by:

(3) $$\begin{array}{l} p\left(k\right)=c \end{array}$$

where *c* is a constant.

*Random networks* (Figure 2Bd) show each node with a different connectivity degree and the probability that a single unit has *k* connections is modeled by a Poisson distribution:

(4) $$\begin{array}{l} p\left(k\right)\propto \frac{e^{-\delta }\delta^{k}}{k!} \end{array}$$

where δ is the average connectivity degree of the network. The random graph has few local connections and therefore it shows low segregation values. The integration levels of the network, instead, follow the logarithm of the number of nodes.

A last case is the *small-world network* (Figure 2Bc): it shares the same characteristics of regular and random networks, constituting a sort of composite model. By increasing the probability *p* of rewiring, the order of a regular lattice is disrupted, and when *p* = 1 a random graph is generated. Increasing the probability of rewiring, both the integration and the segregation levels decrease. In a small-world network, the distance between two nodes grows according to the logarithm of the number of nodes of the graph (Watts and Strogatz, 1998).

As stated before, to characterize the topological features of a network, we need some quantitative metrics. Here below, we introduced three statistics: Cluster Coefficient, Average Path Length, and Small-World Index.

**Cluster Coefficient:** let *i* be a generic node and *u*_*i*_ the spatially nearest nodes to him (called “neighbors”); let $\frac{k_{i}\left(k_{i}-1\right)}{2}$ be the edges that exist among all units within the neighborhood. The connectivity density index of the topological neighbors of this node is the Cluster Coefficient (*C*_*i*_) defined as follows:

(5) $$\begin{array}{l} C_{i}=\frac{\#\;of\;edges\;between\;neighbors\;of\;i}{\frac{k_{i}\left(k_{i}-1\right)}{2}} \end{array}$$

where *k* is the number of connections.

The Average Cluster Coefficient, a global metric often used to quantify the segregation at network level, will be obtained by computing the average of all Cluster Coefficients of each node (Figure 2A, Modules 3 and 4).

**Average Path Length:** let us to consider two generic nodes *i* and *j* of a network *V*. Let *d*(*i, j*) be the shortest distance between *i* and *j*. The Average Path Length (*L*) is defined as follows:

(6) $$L=\frac{2}{n\left(n-1\right)}\sum_{i\neq j}d\left(i,j\right)$$

where *n* is the number of nodes in *V*. This topological measure can be used to evaluate the network's level of integration (Figure 2A, Modules 3 and 4).

Finally, to detect the emergence of small-world network in Downes et al. (2012) combined these metrics, defining the Small-Word Index (*SW*) as:

(7) $$\begin{array}{l} SW=\frac{\frac{C_{real}}{C_{lattice}}}{\frac{L_{real}}{L_{RND}}} \end{array}$$

where *C* and *L* of experimental data (*C*_*real*_ and *L*_*real*_) are normalized against the expected values (*C*_*lattice*_ and *L*_*RND*_) from an equivalent population of random networks with the same number of nodes and links.

The next section will describe how to extract the topological structures and how to study the emerging functional connectivity of neuronal assemblies coupled to MEAs.

## Different types of connectivity to describe neuronal assemblies

Three types of connectivity are used to describe the interactions of neuronal networks: structural, functional and effective.

### Structural connection (Figure 3A)

The *structural* or *anatomical* connection indicates the physical interaction (i.e., a chemical or electrical synapse) that links a network's neurons at a given time (Sporns and Tononi, 2002). Therefore, we can determine which neural units can directly interact with each other. The structural connectivity ranges over multiple spatial scales, since we can detect morphological connections both in local microcircuits and in long-range interactions that link different sub-networks. In a short time scale (about., less than 1 min), such morphological connections mediated by dendritic spines are static, while in a longer time scale, they are dynamic, since physiological mechanisms of learning, plasticity and development can shape the morphological circuits (Buchs and Muller, 1996).

**Figure 3:**
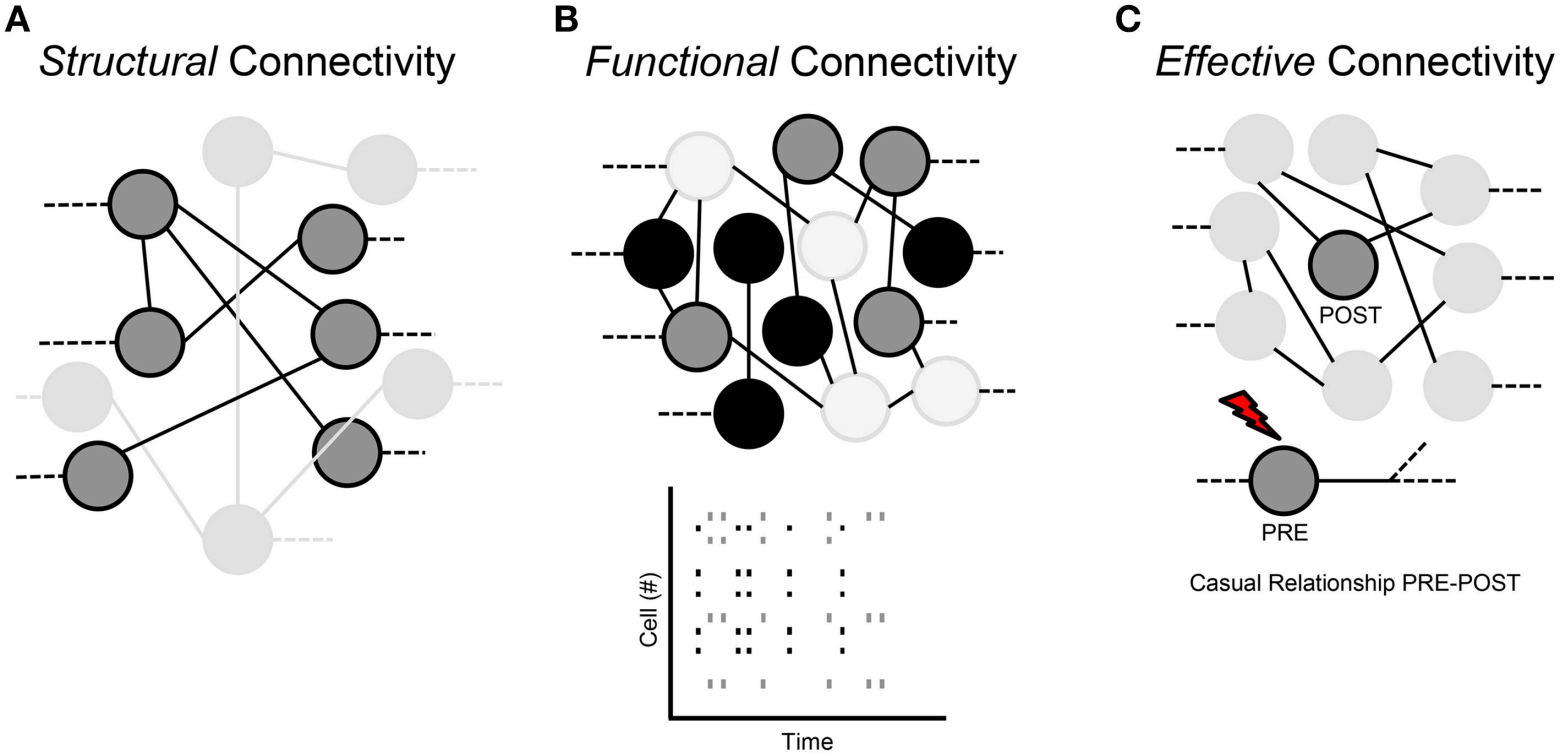
**Classification of the neural network connections. (A)** Structural connectivity. **(B)** Functional connectivity. **(C)** Effective connectivity.

### Functional connection (Figure 3B)

Functional connection indicates the correlation between time series of spikes coming from different neurons. It measures statistical interdependence without considering any causal effects; it is time-dependent and “model-free.” Therefore, two neurons are functionally linked, if we can predict the activity of one of the two neurons on the basis of the activity of the other neuron: this means that functional connections are a subset of the structural ones (Sporns and Tononi, 2002). Indeed, functional properties of single neurons are strongly driven by their anatomical connections, dendritic arborizations and synaptic distributions. Moreover, functional interactions can contribute to the shaping of the underlying anatomical substrate through activity-dependent synaptic modifications.

### Effective connection (Figure 3C)

Effective connectivity indicates the presence of a connection when a neuron on the network directly affects another neuron through a causal relationship between the activities of the two neurons. In other words, “effective” means any observable interactions between neurons that alters their firing activity; so it is not “model-free” like functional connectivity, but can require the specification of a causal model including structural parameters.

## Functional connectivity methods for *in vitro* networks

To estimate the functional connectivity of *in vitro* networks, there are two different strategies: the first one relies on the direct analysis of the acquired sequence of voltage values (Figure 1A top) from each recording electrode (i.e., the time series). The other approach deals with point processes (e.g., spike trains). Practically, a spike train is a sequence of samples equal to 1 if a spike is detected in that sample and 0 otherwise (Figure 1A bottom). The identification of the peaks from the time series can be performed in several ways, ranging from simple spike detection (Maccione et al., 2009; Ide et al., 2010) to spike sorting techniques (Egert et al., 2002) up to more complex multivariate approaches (Borghi et al., 2007).

In the literature, there are several works dealing with the connectivity methods that can be used to infer the functional connectivity of neural networks (e.g., Cutts and Eglen, 2014). The aim of this work is not to describe all the connectivity methods, but rather to show which information is possible to extract from such an analysis applied to *in vitro* neural networks coupled to MEAs. However, to help the reader understanding the results provided in Section Applications, we briefly introduce two widely used algorithms belonging to the family of the correlation methods: Cross-Covariance (CCov) and Cross-Correlation (CC).

### Cross-correlation

Cross-Correlation (CC) is applied to point processes (e.g., spike trains). It measures the frequency at which one cell called “target” fires relative to the firing time of a spike in another cell known as “reference” (Salinas and Sejnowski, 2001). Mathematically, the Cross-Correlation function represents the average value of the product of two random processes, which in this case are the spike trains (Knox, 1981), and it's evaluated considering all the possible pairs of spike trains extracted by the active electrodes. Moreover, connection strength among neurons is evaluated on the basis of the peak values of each Cross-Correlation function and the directionality is derived from the temporal position of the corresponding peak latency. Cross-Correlation reduces to a simply probability *C*_*xy*_(τ) of observing a spike in *y* at time (*t*+τ), if there has been a spike in *x* at time *t* (Rieke et al., 1997); τ is called time shift or time lag. In this context, it is important to take into account the cross-correlogram, which is a temporal function that combines the firing information of one target neuron to a reference one. The cross-correlogram *C*_*xy*_(τ) is computed by counting the spikes in *y* and *x* inside a specific time window ±*T*. The values used for the time shift τ depend on the kind of analysis. To solve intra-neuronal signal propagation (i.e., the propagation of an action potential along the arborizations of the same neuron), a thin time lag is necessary (e.g., 0.1–0.5 ms): these values are consistent with the presynaptic propagation speed (Bonifazi et al., 2005). On the other hand, if the inter-neuronal propagation (i.e., signal propagation mediated by the synaptic transmission) has to be characterized, a wider time shift value can be used (0.8–1.2 ms).

To obtain the maximum correlation peak between 0 and 1, it is possible to normalize *C*_*xy*_(τ) as follows:

(8) $$\begin{array}{l} C_{xy}\left(\tau \right)=\frac{1}{\sqrt{N_{x}N_{y}}}\sum_{s=1}^{N_{x}}\sum_{ti=\left(\tau -\frac{\Delta \tau }{2}\right)}^{\left(\tau +\frac{\Delta \tau }{2}\right)}x\left(t_{s}\right)y\left(t_{s}-t_{i}\right) \end{array}$$

where *t*_*s*_ is the duration of each spike in train *x*, *N*_*x*_ is spike's total number in *x* and *N*_*y*_ represents the spike's total number in *y*. In particular, when two spike trains are independent, the cross-correlogram is flat; if there is any co-variation, one or more peaks appear (Brody, 1999). By considering the peak amplitude of each Cross-Correlation function, we define a Connectivity Matrix (CM) whose highest values are supposed to correspond to the strongest connections. Moreover, the Cross-Correlation function is symmetric since *C*_*xy*_(τ) = *C*_*yx*_(−τ). By exploiting this mathematical property, many of the parameters to extract from the cross-correlogram are symmetric and the computation can be faster (only half of the Cross-Correlation matrix has to be computed).

### Cross-covariance

Cross-Covariance (CCov) is applied to time series data (e.g., *X* and *Y*). We define the Cross-Covariance as the probability to observe a spike in *X* at time *s* and a spike in *Y* at the same time t. This probability is defined as following:

(9) $$\begin{array}{l} CCov\left(s,t\right)=Cov\left(X_{s},Y_{t}\right)=E\left[\left(X_{s}{-\;\mu }_{s}\right)\left(Y_{t}{-\;\mu }_{t}\right)\right] \end{array}$$

where μ_*s*_ and μ_*t*_ are the mean functions defined as *E*[*X*_*s*_] and *E*[*X*_*t*_] respectively.

In the stationary case, Cross-Covariance will be a function of the time lag τ, and can be approximated as:

(10) $$\begin{array}{l} Cov\left(X_{0},Y_{t}\right)=Cov\left(X_{0+\tau },Y_{t+\tau }\right)=C\left(\tau \right) \end{array}$$

Meaning that the Cross-Covariance reduced to the Cross-Correlation in the stationary case. Cross-Covariance shares all the properties described for the Cross-Correlation (e.g., the symmetry). Finally, also the maximum Cross-Covariance value is used as an indication of the strength of functional connection between neurons (Downes et al., 2012). The time series analyzed with the Cross-Covariance approach may be acquired not only by *in vitro* models but also by others techniques that will not be discussed in this work, such as electroencephalography (EEG), magnetoencephalography (MEG), and functional magnetic resonance imaging (fMRI) (Babiloni et al., 2009).

### Connectivity maps

The aforementioned algorithms enable building a CM. The CM is a *n x n* matrix (where *n* is the number of analyzed electrodes) whose generic element (*i,j*) is the estimation of the strength of connection between electrodes *i* and *j*. In detail, the generic element (*i,j*) of the CM is the peak (i.e., the maximum value) extracted from the Cross-Correlation or Cross-Covariance between the electrodes *i* and *j*. Moreover, both Cross-Covariance and Cross-Correlation allow the determination of the direction of the connections. This information is stored in the position of the peak with respect to the central bin of the correlation window. In detail, given the cross-correlogram between electrodes *i* and *j* obtained through one of the aforementioned methods, if the peak is temporally after the central bin, the electrode *i* is presynaptic with respect to the electrode *j* and vice versa (if the peak is placed in the central bin, no information can be extracted on the direction of the connection).

Since the CM can be a full matrix of *n*^2^ elements, a thresholding procedure is required to throw away those values that are close to or in the noise, and not real connections. This requires setting a threshold for the connectivity matrix (TCM). Exploring the available works in the literature about the analysis of functional connectivity of *in vitro* neural networks, it is possible to see several thresholding procedures, with different levels of complexity. The simplest of such procedures, is to use a hard threshold defined as (μ+*n*·σ), where μ and σ are the mean and the standard deviation computed among all the CM's elements, respectively, and *n* is an integer. Another possibility is to use shuffling techniques, that allow to destroy the information stored in the spike timing, obtaining independent spike trains (i.e., surrogate data). A simple application of this technique is presented in Maccione et al. (2012), where only the spike trains relative to a defined number of the strongest connections have been shuffled.

It is worth noticing that there are more sophisticated and complex approaches to obtain surrogate data from the spike trains and to threshold the CM; however, the description of these techniques is out of the scope of this review. For further information we suggest the reading of Grun and Rotter (2010) and references therein. Summarizing, the simplest way to obtain the TCM and analyze the results in functional connectivity analysis of *in vitro* neural networks is to use a hard threshold. However, this thresholding procedure is strongly dependent on the distribution of the CM's values. Shuffling techniques are more precise and less heuristic, but they are computationally heavy. Thus, when dealing with the problem of thresholding the CM, it is important to choose the best compromise between reliability and computational time, depending on what one wants to claim from that specific analysis.

## Applications

**Figure 4:**
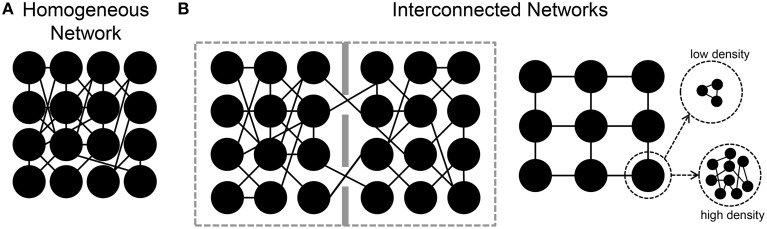
**Sketches of different *in vitro* neuronal assemblies. (A)** A homogeneous network in which neurons are free to connect without any chemical/mechanical constraint. **(B)** Interconnected neuronal networks. *Left*, two small populations are connected by means of a few number of links. *Right*, patterned networks where each node can be a small or large number of neurons.

In the following sections, we will review some results regarding the estimation of the functional connectivity in neuronal assemblies coupled to MEAs. In particular, starting from the analysis of the functional connectivity inferred in large-scale homogeneous neuronal networks (Figure 4A), we then consider the case of engineered networks, where by means of physical or chemical constraints, the structural connections are driven to form interconnected networks (Figure 4B). Finally, we will consider the effects of different patterns of electrical stimulation delivered to the networks to shape the functional connectivity.

### Connectivity and dynamical states

*In vitro* neural networks coupled to MEAs display a spontaneous activity characterized by the presence of spikes and bursts (Figure 1A) in a ratio depending on the stage of development (Wagenaar et al., 2006). Starting from the second week *in vitro* (WIV), dissociated neuronal assemblies show sequences of bursts that give rise to an activity persisting for the entire network life time (Marom and Shahaf, 2002). During the maturation phases (3rd-4th WIV), theoretical and experimental analysis (Abbott and Rohrkemper, 2007; Pasquale et al., 2008) have highlighted periods of increased activity, called *neuronal avalanches*, supporting the evidence of *criticality* in *in vitro* dissociated neuronal networks. Experimental and computational studies proved that a critical system, like a neuronal assembly, maximizes its computational properties by optimizing the information processing (Shew et al., 2011). As reviewed by Hesse and Gross (2014) the use of the Self-Organized Criticality allows to connect the microscopic and macroscopic levels of investigation of a neuronal system. In addition, the use of this theory allows to recognize possible pathologies (e.g., epilepsy) of the brain which disrupt this equilibrium point (Massobrio et al., 2015a). Such a critical state is at a boundary between other types of dynamics (sub-criticality and super-criticality). Indeed, as found in Pasquale et al. (2008), Tetzlaff et al. (2010) some cultures evolve toward a critical state, but several others tend toward sub-critical or super-critical states. The network connectivity organization is one of the possible factors that can drive the network toward a peculiar dynamic state (i.e., critical, sub-critical, super-critical). Recent computational studies claim that a critical state can be sustained if the network organization (both functional and morphological) presents complex features. Pajevic and Plenz (2009) found that both random and small-worlds networks were able to promote critical dynamics in cortical networks. More recently, Massobrio et al. (2015b) proved that different topologies of connectivity induce different dynamic states by pushing the network from sub-critical, to critical, up to super-critical states. In particular, the synthetic results display the existence of a tight interplay between the exhibited dynamics and the topology. Random networks only show super-critical dynamics in a physiological domain of their firing regime. On the other hand, scale-free and small-world architectures account for the variability observed in experimental data and the transition from sub-criticality to criticality is ruled by the degree of “small-worldness.”

**Figure 5:**
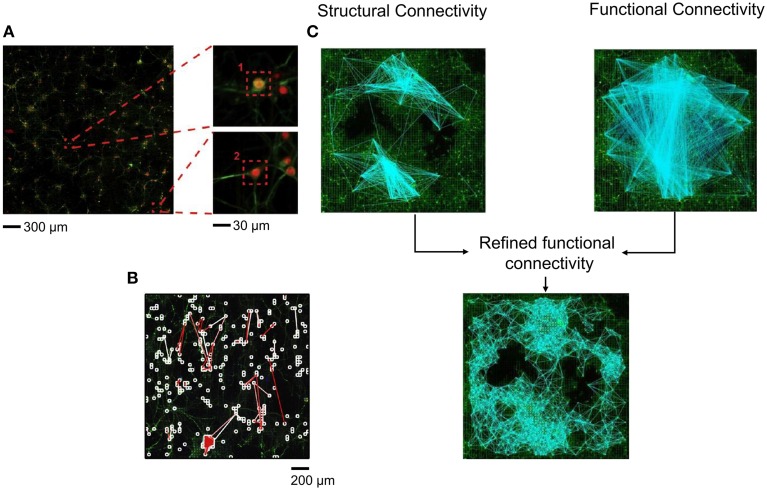
**Structural-functional connectivity analysis on high-density (HD) MEA. (A)** Fluorescence image of a neural culture on the HD-MEA and a zoom at the single neuron level. **(B)** Functional links superimposed on a fluorescence image of a HD-MEA chip. White squares indicate the neurons more strongly connected, while white and red branches represent the links among the identified neurons (Adapted from Maccione et al., 2012). **(C)** Structural connectivity graph reconstructed using imaging methods combined with the functional connectivity graph obtained by Cross-Correlation analysis to obtain a refined functional connectivity graph (Adapted from Ullo et al., 2014).

Most of the indications regarding the kind of topological organization of these dissociated networks emerges from computer simulations. This is mainly linked to the difficulties of determining the network topology of cultures from a limited number of recording sites (60/120 microelectrodes) with a low spatial resolution. In Maccione et al. (2012), the authors analyzed hippocampal cultures at low density (80–200 neurons/mm^2^) recorded by a high density CMOS-MEA, made up of 4096 microelectrodes (Berdondini et al., 2009) able to provide simultaneous multi-site acquisition at high-spatial (21 μm inter-electrode separation) resolution. The use of such a high-density MEA with low-density cultures has allowed mapping neuronal signaling in large-scale networks at spatial resolution down to the cellular level up to a possible identification of its anatomical connections; moreover, it allows the comparison of the inferred effective links with the network structure obtained by staining procedures (Figure 5A). The authors focused on the estimation of functional connectivity from extracellular electrophysiological recordings by applying the cross-correlation algorithm on the acquired spike trains and additional spatio-temporal filtering procedures, that were used to discriminate between possible causal relationships and spurious connections, and thus to improve the reliability of the estimated maps. Finally, they superimpose the functional-effective detected links to fluorescent morphological images of the cultures, combining structural and functional information (Figure 5B). They found that the strongest functional connections corresponded to the shortest path length; this information, together with visual comparison with the morphological image, suggested that possibly direct synaptic connections were identified. More recently, in Ullo et al. (2014), the authors focused on the investigation of the tight interplay between structural and functional connectivity, combining high-resolution functional data acquired with the HD-MEA with fluorescence microscopy imaging. Such an approach can enable the unprecedented mapping of both activity and structure of neural assemblies at a cellular level. The authors hypothesized that the presence of a strong structural connection makes a functional connection more likely to occur. Thus, they localize neurons with respect to the electrode array and estimate the structural connectivity using imaging methods; finally, the structural connectivity graph was used as a prior to refine the functional connectivity estimated through a Cross-Correlation analysis (Figure 5C), obtaining a more realistic and less connected network graph. However, despite the combination of structural and functional information, no analysis has been done on the topological parameters determination.

### Functional connectivity during development

**Figure 6:**
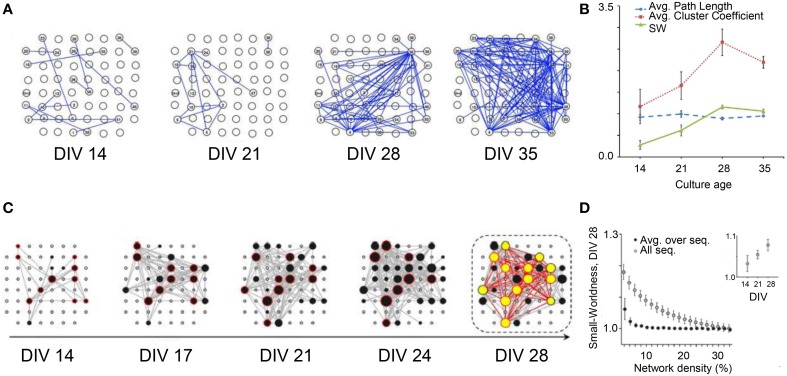
**Topological network properties during development. (A)** Organization of the network structure at different stages of development (DIV: 14, 21, 28, 35). **(B)** Average Path Length (blue line), Average Cluster Coefficient (red line) and Small-Worldness curve (green line); the increase of the small-worldness curve during development, evaluated as (*C*_*real*_*/C*_*lattice*_)/(*L*_*real*_*/L*_*rand*_), shows a significant reorganization of the network (*p* < 0.05), from a random structure to a small-world architecture (Adapted from Downes et al., 2012). **(C)** Functional connectivity during the first 4 weeks *in vitro*; the hubs (black and yellow dots) promote the small-world topology; the number and the connection degree of the hubs increase during development. **(D)** Small-Worldness decreases with network density from 10 to 30 (DIV 28), but increases during the first 4 weeks *in vitro* (Adapted from Schroeter et al., 2015).

In 2012, the research group led by Nasuto characterized the evolution of the functional topological features of *in vitro* cortical assemblies during development (Downes et al., 2012). The authors demonstrated the emergence of small-world functional properties during the development of spontaneous activity. In particular, they characterized the connectivity graphs extracted from cultures during the first 5 weeks *in vitro* (Figure 6A) by evaluating the degree of segregation and integration. This analysis was done by applying Cross-Covariance to the raw data (i.e., time series) for evaluating the Average Cluster Coefficient, the Average Path Length, and the Small Word Index, respectively (cf. Section Graph Theory). Young cortical cultures (14 days *in vitro*, DIV) started to fire with a random connectivity (low values of both measures). However, during development, the functional connectivity changed and the topological features of the networks evolved toward a small-world topology. Figure 6B shows an increase of the Average Cluster Coefficient with age (red line), keeping low and constant the Average Path Length values (blue line). The SW index (cf. Section Graph Theory) showed, therefore, a significant reorganization of the network from a random structure to a small-world architecture (green line).

Another proof of the emergence of small-world topology during development has been recently provided by Schroeter et al. (2015). The authors worked on the time series acquired from hippocampal *in vitro* neural assemblies, using Cross-Covariance to infer functional connectivity. Differently from Downes et al. (2012) they applied Cross-Covariance not to the entire recorded activity, but only to the detected network bursts. The authors found the presence of highly connected nodes (i.e., hubs) starting from DIV 14. In addition, they identify a Rich-Club topology, that is the presence of hubs more densely interconnected with each other than expected by chance (Colizza et al., 2006), leading them to discard the random topology hypothesis (Figure 6C). Even if both Schroeter et al. (2015) and Downes et al. (2012) showed the emergence of a small world topology (Figures 6B,D respectively), only Schroeter and coworkers found the presence of such a Rich-Club organization, in agreement with recent *in vivo* results, demonstrating that the structural network of the human brain presents a “rich-club” organization (Van Den Heuvel and Sporns, 2011). These different results could be partially explained by the different cell density at which cultures are seeded. Indeed, dense cultures mature faster than their sparse equivalents (Wagenaar et al., 2006); Downes and coworkers plated at a cell density of (950–3750) cell/mm^2^, while Schroeter and coworkers used a density much lower (180–440) cell/mm^2^.

The changes in functional connectivity during development have been also analyzed by Napoli et al. (2014). However, differently from the previous works, they investigated network changes of dissociated cortical neurons focusing on network responses within selected time windows (50, 100, 150 ms) after stimulus sessions, quantifying the temporal evolution of the neural population activity through the False Discovery Rate (FDR) technique. FDR is a statistical significance test that measures how similar two different distributions are (Napoli et al., 2014); it is defined as $E\left[\frac{V}{R}\right]$, where *V* and *R* are the number of false connections and the total number of connections, respectively. Differently from Downes et al. (2012), Napoli et al. did not focus on the network topology, but on the connection length variability that they found significant among different batches during network development by means of statistical analysis of stimulus-evoked response dynamics, emphasizing the importance of the time window choice.

### Functional connectivity in engineered networks

In the examples discussed in the previous sections, neurons were spread homogeneously over the MEA surface, and free to grow and establish synaptic connections without any kind of constraint. As the sketch of Figure 4A shows, they recreate a dense homogeneous network. Indeed, to have more “connectivity-controlled” networks, and closer to the actual *in vivo* segregation/integration of the brain, several attempts have been performed to “design” engineered networks where the connectivity is (partially) controlled (Macis et al., 2007; Baruchi et al., 2008; Fuchs et al., 2009; Kanagasabapathi et al., 2011; Shein-Idelson et al., 2011; Marconi et al., 2012; Pan et al., 2015). In most of these works, the goal was to design interconnected or spatially segregated networks (Figure 4B), where the different neuronal populations could present different sizes (e.g., from a few (Marconi et al., 2012), to tens (Macis et al., 2007) to hundreds (Kanagasabapathi et al., 2011) of neurons), different neuronal populations (e.g., cortical-thalamic networks, Kanagasabapathi et al., 2012), or different number of modules (Berdondini et al., 2006; Kanagasabapathi et al., 2011).

**Figure 7:**
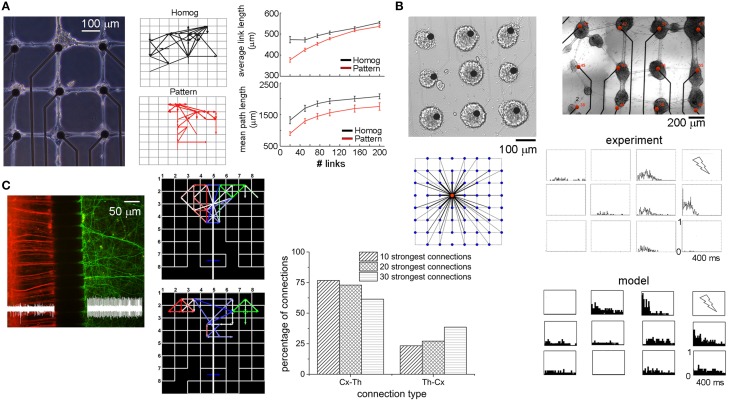
**Examples of engineered neuronal networks. (A)** *left*, Example of a bio-patterned network aligned with the electrode array; *middle*, two examples of functional connectivity maps relative to a homogeneous (black) and a patterned (red) network; *right*, average link length and mean path length for homogeneous (black) and patterned (red) cultures as a function of the number of strongest links (Adapted from Marconi et al., 2012). **(B)** Two examples of patterned networks realized with a micro-drop delivery system; *bottom-left*, connectivity map governing the connectivity of this patterned network; right, PSTH maps relative to one experimental (top), and one simulated phase (bottom) (Adapted from Macis et al., 2007; Massobrio and Martinoia, 2008). **(C)** Cortical–thalamic co-culture plated in a dual compartment device (cortical cells and thalamic cells are highlighted with red and green fluorescence staining, respectively). *Middle*, two examples of functional connectivity maps related to a cortical-thalamic system. Red, green, and blue links refer to cortical-cortical, thalamic-thalamic, and cortical-thalamic connections, respectively. *Right* distribution of the inter-cluster connection in a cortical-thalamic system (Adapted from Kanagasabapathi et al., 2012).

In 2012, Marconi et al. (2012) developed a bio-printing method to design the topology (and thus drive the connectivity) of *in vitro* hippocampal cultures (Figure 7A, left panel). The authors coupled the micro-contact printing of an adhesion promoter with the use of an agarose repulsive layer and investigated the emergent functional connectivity compared to homogeneous neuronal cultures. By applying a Cross-Correlation function to the spike trains of patterned and homogeneous hippocampal cultures, they extracted functional connectivity maps (two examples are reported in Figure 7A middle panel). The connectivity matrix was thresholded by sorting the strongest links. The “quasi-regular” topology induced a reinforcement of functional connections along orthogonal directions, shorter connectivity links and a greatly increased spiking probability in response to focal stimulation. The top right panel of Figure 7A compares the average link lengths of homogeneous (black lines) and patterned (red lines) networks. Patterned networks present shorter links than found in the homogeneous ones. This link length difference is relevant only when a few number of links (less than 100), are taken into account: in other words, when the strongest functional connections are considered, patterned networks present shorter links than the ones detected in homogeneous networks. When the number of links is higher (meaning that a low threshold has been chosen) such a gap decreases, suggesting the importance (and the dependence) of the threshold selection in this kind of measures (cf. Section Connectivity Maps). Another interesting result regards the degree of clusterization of these networks: although the clustering coefficient was low and comparably in both patterned and homogeneous cultures, the mean path length was always (i.e., independently of the number of considered links) lower in the patterned topology (Figure 7A, bottom right panel, red line) than in the homogeneous one (black line). This result should enhance the efficacy of propagation of the electrical activities among the neurons of the network. The complexity of the patterning procedure (i.e., the maximum number of connections that each node can establish) strongly shapes the network dynamics (Boehler et al., 2012). Although the firing rate results comparable between homogeneous and patterned networks, burst duration monotonically increases as a function of the complexity of the network circuitry (Boehler et al., 2012), suggesting that longer bursts might result from networks that integrate several synaptic pathways (both inhibitory and excitatory).

The possibility to drive the connectivity among neurons was also pursued some years before by Macis and coworkers who developed a micro-drop delivery set-up based on a piezo-dropper system and a motorized *X–Y* stage which allows the deposition of small volumes (about 100 pl) of specific adhesion molecules on the MEAs (Macis et al., 2007). As the top panels of Figure 7B show, neurons were anchored on the substrate only in the areas where the adhesion proteins have been deposited, defining high density sub-networks (about 4'000 neurons/mm^2^) interconnected by means of bundles of neurites. The topological rule existing among these clusters of neurons was estimated by means of a computational model (Massobrio and Martinoia, 2008) whose connectivity rule is depicted in the left bottom panel of Figure 7B. The authors demonstrated that the dynamics displayed by the considered patterned neuronal networks could be explained by hypothesizing the presence of several short- and few long-range interactions among the small assemblies of neurons. The matching between the experimental recordings and the *in silico* results was achieved by comparing both the spontaneous and the stimulus-evoked activity. The bottom right panels of Figure 7B display the Peri-Stimulus Time Histogram (PSTH) profiles when the culture in the top panel is stimulated with a low-frequency (0.2 Hz) bi-phasic 1.5 V voltage stimulus delivered to the top-right electrode. As it might be expected, the electrodes show site-specific responses reflecting the functional topographical connectivity of the network: the closest electrode to the stimulating site shows a fast response, while as far as the distance increases a more delayed response appears. In addition, some electrodes displayed an attenuate response to the stimulus or, in some cases, no evoked activity. Some years later, Ide et al. (2010) found in homogeneous hippocampal networks that the probability of evoking a response decreases with a *quasi* linear relationship with the distance. The *in silico* model well reproduces such a behavior: the closest neurons to the stimulation site present a fast and marked response, whereas the others can present a more delayed and attenuate response, or in some case, no evoked activity.

An intermediate scenario between large-scale homogeneous assemblies and patterned/ordered networks is the dual compartment system devised by Kanagasabapathi et al. (2011). By coupling a Poly-dimethyl-siloxane (PDMS) mask to the surface of a MEA, the active area was divided in two sub-regions interconnected by means of an array of micro-channels (3 μm height, 10 μm width). The small height of these micro-channels prevents the movement of cells between compartments while a length greater than 100 μm selects for axons (Morales et al., 2008) to cross-over to the adjacent compartment and form a functional network. Figure 7C (left) displays a possible application of this system for co-culturing heterogeneous neuronal populations like cortical (red) and thalamic neurons (green) to study the reciprocal interactions in terms of dynamics and connectivity (Kanagasabapathi et al., 2012). The interplay between cortico-thalamic and thalamo-cortical populations was investigated, by estimating the functional connectivity between the two populations by computing Cross-Correlation on the spike trains. Two examples of functional connectivity maps evaluated by considering the strongest 20 intra-cluster and 10 inter-cluster connections are shown in the left bottom panel of Figure 7C. Direction of the links was derived by the peak latency of the cross-correlogram. The authors found that ~77% of the connections are cortico-thalamic, while ~23% was thalamo-cortical. By varying the number of connections (i.e., from 10 to 30), an increase in the fraction of thalamo-cortical links was observed indicating that the strongest connections are from cortical to thalamic population (Figure 7C, bar plot). This reciprocal connectivity between the two neuronal populations explains the emergent dynamics: burst events originate in the cortical region and the presence of strong cortico–thalamic connections drives the thalamic network to discharge bursts while reciprocal weak thalamo-cortical connections play a salient role in the cortical network behavior by modulating the duration and shape of the burst event.

### Shaping the connectivity by electrical stimulation

The use of dissociated neuronal cultures coupled to MEAs allows the design of experiments where neurons can be extracellularly stimulated by means of electrical pulses delivered through the same electrodes of the device. In this way, it becomes reasonable to investigate how the emerging neuronal dynamics can be modulated by the electrical stimulation and, consequently, whether the underling functional connectivity is modified or not. Several studies report that depending on the features of the electrical stimulation (i.e., number of stimulated sites, frequency stimulation, amplitude of the pulse, etc.) the network activity can evolve toward new dynamical states. The hypothesis that certain patterns of activity can change synaptic efficacy is a recognized milestone (Shahaf and Marom, 2001; Eytan et al., 2003; Bakkum et al., 2008).

**Figure 8:**
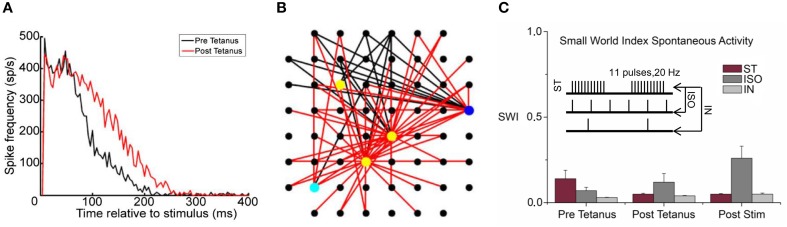
**Effective and functional connectivity analysis. (A)** PSTHs showed a network synaptic potentiation during evoked responses after the tetanus delivery (black and red lines indicate the phases before and after tetanus, respectively). **(B)** Map of the effective connections: a huge increase of the connections (red and black links correspond to the post- and pre-tetanus connections respectively) was found between pre- and post-tetanus phases, explaining the potentiation effect of the network. **(C)** Emergence of a random structure during spontaneous activity, the histogram shows the low SM index values evaluated for three different stimulation protocols (tetanic stimulation without (ST) or with a 0.2 Hz low-frequency (IN) in phase or 1 Hz iso-frequential (ISO) co-activation, inset) and for each recording phases (**A,B** Adapted from Chiappalone et al., 2008).

In 2008, Chiappalone et al. (2008) found that the application of a high frequency tetanic stimulation without (ST) or with a 0.2 Hz low-frequency (IN) in phase or 1 Hz iso-frequential (ISO) co-activation was able to induce a global network synaptic potentiation. The PSTHs of Figure 8A show the network response before (black line) and after (red line) the tetanus delivery. The network response clearly increased because of a synaptic potentiation that can be appreciated by looking at the increase of the number of the effective connections of the network (Figure 8B, red vs. black lines). In addition, it was found that the functional topological structure did not change during the spontaneous activity of neuronal networks. Low SW index values and weak statistical differences among them (Figure 8C) suggest a random network architecture. This result supported the initial hypothesis that external electrical stimulations increase or stabilize the integration rather than segregation processes during spontaneous activity.

In 2010, Le Feber et al. (2010) tried to find a correlation between neuronal connections and slow-frequency stimulation protocols able to induce synaptic changes. They applied to cortical cultures in the mature stage of development biphasic current pulses at a frequency of 0.2–0.33 Hz) to investigate possible modifications on the network functional connectivity, and consequently synaptic efficacy. In addition, the authors investigated the relevance of the stimulation site, by delivering such low frequency pulses both from one site and from different randomly chosen sites. They found that electrical stimulation (independent of the stimulation sites) affects the number of functional links, as well as the average magnitude of changes. However, although the stimulation site does not affect the variations of connectivity, it is worth noting that the delivery of a stimulus from one electrode does not necessary induce the same functional connectivity changes when the network is stimulated from another one. Only the magnitude of changes were preserved. The weak point of this work is that the authors did not make any claim about possible changes in the topology of the networks induced by the stimulation. A change of the number of the functional links, as well as, a change of the efficacy of the links do not necessary mean a change in the topology of the network. To the best of our knowledge, no studies about the interplay between topology and electrical stimulation have been performed. In the light of *in vivo* clinical applications like Deep Brain Stimulation (DBS), understanding whether electrophysiological changes of specific brain regions (providing therapeutic benefits for otherwise-treatment-resistant disorders) are sustained by reversible or irreversible alterations of the topological architecture (Kringelbach et al., 2007) will be a great breakthrough.

## Final remarks

Although the idea that brain functions derive from the interactions among neurons has been accepted for decades, only in the last years has it been possible to estimate the “connectome” (Sporns et al., 2005). Advances in technological development combined with powerful computational data-analysis tools, have accomplished new avenues for understanding the interplay between structure and function of the human brain (Sporns, 2013). The ways to infer connectivity are numerous, since also the definition of connectivity is not unique. As reviewed by Feldt in 2011, three major families of connectivity can be described: structural, functional and effective connectivity (Feldt et al., 2011). These types of connectivity (equally important) reflect three parallel levels of investigation: the anatomical connections, the statistical interdependencies and the causal relationships between neurons belonging to the same network. However, tight interdependencies can be found among these connectivity definitions. As reviewed by Bullmore in 2009, “*direct comparisons of structural and functional connectivity* […] *suggest that structural connections are highly predictive of functional connections.* […] *current evidence suggests that topological parameters are generally conserved between structural and functional networks”* (Bullmore and Sporns, 2009). Thus, the estimation of functional and/or structural connections, at different investigations levels (i.e., *in vitro* and *in vivo* models), is possible. However, independently of the scale of investigation, a common approach can be found: a network can be treated as a graph. In graph theory (Harary, 1969), a network is defined as a set of nodes connected by means of edges. The advantage to treat a neural assembly as a graph is that it can be characterized by applying the metrics used to define the properties of a graph itself. The step immediately before the graph is the definition of the actual connections (structural, functional or effective) of the network. In this work, we reviewed some recent insights regarding the functional connectivity properties emerging from multi-site recordings of *in vitro* neuronal preparations. Although this experimental model is extremely simple, dissociated neuronal assemblies coupled to MEAs are widely used to better understand the complexity of brain networks (Schroeter et al., 2015). Despite recent advances in electrophysiology and imaging, the possibility to investigate specific *in vivo* neuronal circuits is still limited and the extracted connectivity maps difficult to solve and understand because of the three dimensional connectivity. Thus, a simplified, but at the same time valid *in vitro* model is necessary to perform investigations about the emergent connectivities and their interdependences with the observed dynamics (Shein et al., 2009). The use of dissociated cultures, which allows network-engineering and simultaneous multi-site electrical recordings, allows to bridge the gap. In this work, we presented some examples of the use of this experimental model taking into account different experimental protocols (e.g., spontaneous vs. stimulus evoked activity, Downes et al., 2012; Napoli et al., 2014), different neuronal preparations (e.g., homogeneous vs. heterogeneous cultures, Kanagasabapathi et al., 2012) and network layouts (e.g., homogeneous vs. interconnected networks, Marconi et al., 2012) for characterizing the emergent topological properties which sustain the actual dynamics.

### Conflict of interest statement

The authors declare that the research was conducted in the absence of any commercial or financial relationships that could be construed as a potential conflict of interest.
